# Validity of the Empatica E4 Wristband to Measure Heart Rate Variability (HRV) Parameters: a Comparison to Electrocardiography (ECG)

**DOI:** 10.1007/s10916-020-01648-w

**Published:** 2020-09-23

**Authors:** Angela A. T. Schuurmans, Peter de Looff, Karin S. Nijhof, Catarina Rosada, Ron H. J. Scholte, Arne Popma, Roy Otten

**Affiliations:** 1 Department of Research and Development, Pluryn, P.O. Box 53, 6500 AB Nijmegen, The Netherlands; 2 Behavioural Science Institute, Radboud University Nijmegen, P.O. Box 9104, 6500 HE Nijmegen, The Netherlands; 3 Wier, Specialized and Forensic Care Fivoor, Den Dolder, Netherlands; 4 Praktikon, P.O. Box 6909, 6503 GK Nijmegen, The Netherlands; 5 Tranzo, Tilburg University, P. O. Box 90153, 5000 LE Tilburg, The Netherlands; 6 Department of Child and Adolescent Psychiatry, VUmc/De Bascule, P.O. Box 303, 1115 ZG Duivendrecht Amsterdam, The Netherlands; 7 ASU REACH Institute, Department of Psychology, Arizona State University, P.O. Box 876005, Tempe, AZ 85287-6005 USA

**Keywords:** Autonomic nervous system, Electrocardiography, Empatica, Heart rate variability, Validation, Wearables

## Abstract

Wearable monitoring devices are an innovative way to measure heart rate (HR) and heart rate variability (HRV), however, there is still debate about the validity of these wearables. This study aimed to validate the accuracy and predictive value of the Empatica E4 wristband against the VU University Ambulatory Monitoring System (VU-AMS) in a clinical population of traumatized adolescents in residential care. A sample of 345 recordings of both the Empatica E4 wristband and the VU-AMS was derived from a feasibility study that included fifteen participants. They wore both devices during two experimental testing and twelve intervention sessions. We used correlations, cross-correlations, Mann-Whitney tests, difference factors, Bland-Altman plots, and Limits of Agreement to evaluate differences in outcomes between devices. Significant correlations were found between Empatica E4 and VU-AMS recordings for HR, SDNN, RMSSD, and HF recordings. There was a significant difference between the devices for all parameters but HR, although effect sizes were small for SDNN, LF, and HF. For all parameters but RMSSD, testing outcomes of the two devices led to the same conclusions regarding significance. The Empatica E4 wristband provides a new opportunity to measure HRV in an unobtrusive way. Results of this study indicate the potential of the Empatica E4 as a practical and valid tool for research on HR and HRV under non-movement conditions. While more research needs to be conducted, this study could be considered as a first step to support the use of HRV recordings provided by wearables.

## Introduction

The past two decades have witnessed an increase in psychophysiological studies that incorporate heart rate (HR) and other autonomic nervous system (ANS) parameters. In particular heart rate variability (HRV) has become the focus of psychophysiological research since it provides several parameters of the parasympathetic nervous system (PNS; [1]). These parameters serve as an index of an individual’s physiological reactivity to stress. Stress activates the sympathetic nervous system (SNS), responsible for high arousal including the fight-or-flight response, whereas the PNS facilitates the rest and digest response. Both branches are essential for the immediate stress regulatory response of the body [2]. The PNS is associated with self-regulation aspects of cognition, affection, and social behavior [3].

Most traditional devices that measure ANS parameters are based on electrocardiogram (ECG) recordings, such as the Biopac (Biopac ECG Module, Goleta, CA) or the VU University Monitoring System (VU-AMS; Vrije Universiteit, Amsterdam, the Netherlands). The VU-AMS is a lightweight ECG device for ambulatory assessment that is considered to be a ‘gold standard’ [4, 5]. Although the VU-AMS provides excellent opportunities for ambulatory measurements in real-life contexts, application of the electrodes and setup of the device needs to be done by an expert. Simpler and less invasive monitoring systems such as wearable wristbands have been developed as a more convenient way to measure physiological parameters. Recent advances in technology, and in particular the development of wearable monitoring devices, have provided both researchers and lay people with a simple, non-invasive way to measure HR. The new generation of health monitoring devices consists of easy wearable devices that are worn as a smartwatch. Ideally, these wearables are non-intrusive, robust to movement, and highly accurate [6]. The use of these wearable wristbands in healthcare yields high expectations, but it is unclear whether these expectations are warranted [7]. There are several commercially available wristbands that potentially provide a range of HRV parameters, such as the Empatica E4 wristband [7–9], the Polar watch, [10, 11], and the Fitbit watch [12–14] among others. These devices provide a potentially simple and promising tool for data acquisition in both research and clinical studies [15–18], but are artefact prone due to movement [2, 15]. Due to their non-invasive way of monitoring, these devices are in particular suitable for vulnerable populations such as clinical patients.

Although the reliability and validity of the VU-AMS to obtain HRV parameters has been established [4, 5], there is still debate on the validity of wearables as HRV monitoring systems. The use of these wearables in real-life is in particular challenging as there is considerable amount of movement, temperature fluctuation, and diurnal variation in HRV that could influence the recordings and subsequently the utility of the data [2, 16]. Validation studies are critical to ensure the accuracy, reliability and limitations of wearables before recommending their widespread adoption as a research tool. Studies testing the Polar V800 [10, 11] and the FitbitChargeHR™ [12] demonstrated that HR and HRV recordings provided by wearables can be highly comparable and show high agreement with those of ECG systems.

Another type of wearable is the Empatica E4 wristband. Although previous studies suggested that Empatica E4 recordingse are comparable to ECG [8, 9, 19, 20], these studies were no rigorous validation studies and had several limitations. While all compared the Empatica E4 to ECG, none of these studies used an ambulatory gold standard instruments such as the VU-AMS as reference device [4, 5]. Second, despite its potential effect on the detection of stress and emotion [21], only Van Lier et al. [19] provided details about the application of the Empatica E4 wristbands. They attached the Empatica E4 on participants’ left wrists, so they were unable to make a comparison of different measurement conditions (e.g., left/right hand, dominant/non-dominant hand). Third, most of these studies included only a few time-domain ANS parameters such as HR and RMSSD. Only Ollander et al. [9] included frequency-domain measurements too. None of the previous studies included SDNN, although SDNN is considered the best parameter for medical stratification of cardiac risk [22]. Fourth and final, the studies of McCarthy et al. [8], Ollander et al. [9], and Zheng and Poon [20] were conducted with small sample sizes ranging from one to seven participants. Only the study of Van Lier et al. [19] was adequately powered, but their sample consisted of University students only. In applied research, external validity is critical. Because of their non-intrusiveness, wearables are a promising tool for use in clinical research. Yet, it is important to test the validity of these tools not only under ideal circumstances, but also in clinical settings when deployed in under real-life routine conditions [23]. Therefore, the present study aimed to evaluate the accuracy and predictive value of the Empatica E4 wristband by comparing it to the VU-AMS as reference golden standard while worn on both wrists in a clinical population of adolescents in residential care.

## Methods

### Participants

Data for this study were obtained from a feasibility study testing three game-based meditation interventions among adolescents in residential care [24]. This study yielded data of fifteen participants who wore two recording devices during two experimental testing sessions and twelve intervention sessions. During the experimental testing sessions and at the beginning of each intervention sessions, participants’ baseline HRV parameters were measured. The intervention sessions also included at least two measurement moments of participants’ heart rate parameters during short meditation sessions. For a detailed description of the study protocol see Schuurmans and colleagues [25]. The sample consisted of fifteen adolescents (nine males, six females) with a mean age of 14.46 years (standard deviation [SD] = 2.40).

### Sample size

We expected that the recordings of the two measurement devices would be strongly correlated with an effect size of at least .5 [26]. According to the sample size requirements for estimating ICCs proposed by Bonett [27], this would require a sample size of at least 218 cases.

Although our sample did not consist of a large number of individual participants, the study did include multiple measurement days for each participant, as suggested by Bonett [27]. One experimental testing session was conducted before the start of the intervention and one after the intervention ended. During these experimental testing sessions, one recording was conducted. During the twelve intervention sessions, at least two recordings were conducted. Recordings that were retrieved during the sessions took three-to-five minutes. Data from one participant was excluded due to a high frequency of premature atrial complexes (PACs), a common arrhythmia which is considered a benign phenomenon that could impact assessments. Two participants dropped out because they refused to continue with the study. In total, 356 identical segments of NN intervals were recorded, which can be considered sufficient.”

### Procedure

The current validation study used different levels of validity assessment, as suggested by Van Lier et al. [19]. They identified three levels of validity assessment: (1) signal level: the most direct comparison that assesses the capability of a device to generate the same raw data as the reference device; (2) parameter level: whether a device produces physiological parameters (e.g. HR) for each individual similar to the reference device; and (3): event level: a comparison with the reference device on ability to significantly detect event(s) via group means. In the current study, the validity of the Empatica E4 was assessed on the signal level with intraclass correlations (ICCs), cross correlations (CCs) and parameter level with Bland Altman plots. For the current study, no data were available on the event level.

Ethical review and approval were provided by the CMO Arnhem-Nijmegen under protocol NL58674.091.16. Adolescents were recruited within three residential youth care institutions. All participants gave written informed assent and their legal guardians gave written consent. Participants were randomly assigned to one of three conditions: *Muse*, *Daydream*, or *Wild Divine Games*. Although the conditions consist of three different interventions, all make use of meditation-based relaxation techniques and short meditation sessions. Thus, data recordings of the three interventions were highly comparable, making these data suitable for validation of the Empatica E4 wristband. Participants received a 15 euro gift check at the end of the second experimental testing session.

### Data recording

Recordings were conducted at the pre-test experimental sessions (week 1), the intervention sessions (week 2–7), and at the post-test experimental session (week 8). Participants wore two recording devices during all sessions: the Empatica E4 wristband (Empatica Inc., Cambridge, MA, USA; [8, 9, 19]) and the VU-AMS [4, 5]. Baseline HRV parameters were obtained while participants watched an aquatic video. This is a common procedure to achieve a measurement of baseline recordings to which to compare the parameters retrieved during other conditions [28]. Participants were instructed to sit quietly and watch the aquatic video for four minutes. Halfway the intervention there were two participants who refused to continue with the VU-AMS recordings, due to discomfort with the electrodes that needed to be applicated and removed each session. These participants completed the remaining sessions without VU-AMS recordings.

### Empatica E4

**Fig. 1:**
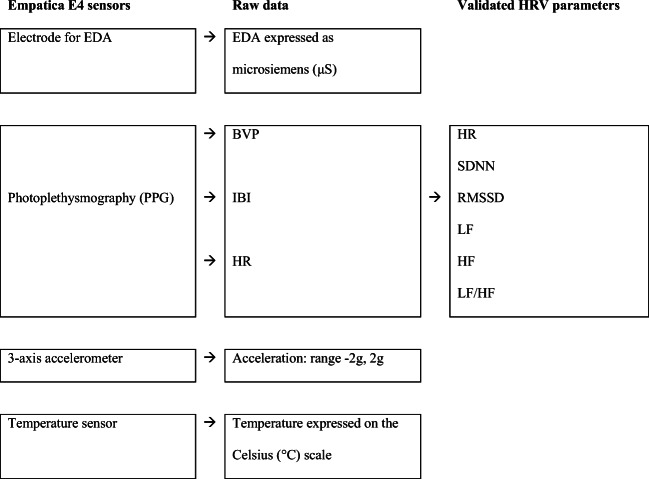
Block diagram for the Empatica E4 wristband. Note. BVP = blood volume pulse, EDA = electrodermal activity, HF = high frequency, HR = heart rate, IBI = inter beat interval, LF = low frequency, LF/HF = ratio between low and high frequency, RMSSD = root mean squared differences of successive difference of intervals, SDNN = standard deviation of the normal to normal interval

The Empatica E4 wristband contains four sensors: (1) an electrode for Electrodermal activity (EDA), (2) 3-axis accelerometer, (3) a temperature sensor, and (4) a photoplethysmography (PPG) to measure blood volume pulse (BVP) from which it derives HR and the inter beat interval (IBI) ([29]; see Fig. 1). Using the Empatica Manager, data were uploaded to Empatica Connect and raw CSV data were downloaded and analyzed using Kubios HRV 3.0 [30]. Kubios offers five artefact correction options based on very low to very high thresholds. We compared Empatica E4 recordings with all five Kubios artefact correction levels to the VU-AMS recordings and without any Kubios artefact correction. Recordings without post-hoc artefact correction showed the highest correlation, so no Kubios artefact correction was used for the analyses. This is not surprising, since the Empatica E4 already uses an algorithm that removes wrong IBIs [31].

### VU-AMS

The VU-AMS is a lightweight ambulatory device that records the electrocardiogram and changes in thorax impedance from seven electrodes placed on participants’ chest and back. Five electrodes are placed on the chest and two on the back. Participants need to partially undress (i.e. lift up their shirt) for placement of the electrodes. The electrodes are connected to a small device that can be worn unobtrusively underneath participants’ clothes. Participants are able to perform their normal daily routines with little constraint in their movements. The ECG had a sampling rate of 1000 Hz and heart rate was obtained from the time between two adjacent R waves. For a detailed description of the VU-AMS assessment procedures see Vrije Universiteit [32]. Heart rate data were extracted and visually inspected for artefacts with the Data Analysis and Management Software (DAMS) program version 4.0.

### Data analysis

Time domain analysis concerns the amount of HRV within the samples. To calculate HRV parameters for time-domain analysis, 343 identical segments of NN intervals were selected from the VU-AMS and E4 recordings. These metrics include:

- RR intervals (RR): the number of detected R waves in the ECG.
- mean HR: average time between two heart beats.
- SDNN: the standard deviation of the NN interval, based on normal sinus beats, thus abnormal beats (e.g. ectopic beats that originate outside the rights artrium’s sinoatrial node) are removed. SDNN tends to be higher when the LF band has more power compared to the HF band [22].
- RMSSD: the root mean squared differences of successive difference of intervals, also based on normal sinus beats. RMSSD stands for HR beat-to-beat variance and is the main estimation for PNS mediated changes in HRV [22].

Frequency-domain analysis allows for estimating sympathetic and parasympathetic contributions of HRV. To calculate HRV parameters for frequency-domain analysis, 243 identical segments of NN intervals were selected from the VU-AMS and E4 recordings (since frequency-domain analysis requires recordings of at least five minutes). Fast Fourier transformation allows for separating HRV into components of the power spectrum:

- Low frequency (LF) activity (0.04 to 0.15 Hz). When measured under resting conditions, like in the present study, it typically reflects baroreceptor activity, which helps to maintain blood pressure [22].
- High frequency (HF) activity (0.15 to 0.40 Hz) reflects PNS activity and is highly correlated with RMSSD [22]. The ratio between low and high frequency power (LF/HF) is an estimation for the ratio between SNS and PNS activity. LF/HF might provide insight in the relative influence of the SNS and PNS, but there is debate on the relative relationship of both branches [15].

### Statistical Analysis: Accuracy

Descriptive statistics (mean and SD), intraclass correlation (ICC) and cross-correlations (CC) were calculated for all variables. Cross-correlations of > .80 were considered valid [19]. Normality was assessed by Kolmogorov-Smirnov tests. None of the variables were normally distributed (all *p* < .05). Mann-Whitney tests were used to detect differences between VU-AMS and E4 recordings. Effect size values (*r*) were calculated for the significantly different outcomes to determine the effect sizes [26]. Difference factors (DF%) were calculated to give a difference estimation in terms of percent (X_VU-AMS_ – X_E4_) / X_VU-AMS_ as was done by Ollander et al. [9]. Bland-Altman plots were constructed and 95% limits of agreement (LoA), where the true value varies, were calculated for all parameters [33]. Bland-Altman plot analysis provides an evaluation for the bias between mean differences of two methods, and an estimation for an agreement interval wherein 95% of the differences of the second method fall, compared to the first.

### Statistical Analysis: Predictive Validity

To evaluate predictive validity, it was assessed to what extent recordings provided by the Empatica E4 wristband led to the same conclusions as the VU-AMS. We conducted analyses to assess potential differences between the three game-based interventions. For each condition, Mann-Whitney tests were conducted to test whether ANS parameters that were recorded during meditation could be distinguished from those recorded during rest.

All analyses were conducted four times: with Empatica E4 recordings of the device worn on participants’ left hand, worn on participants’ right hand, worn on participants’ dominant hand, and worn on participants’ non-dominant hand. For parsimony, only data of the Empatica E4 recordings on participants’ left hand are reported. Differences with the E4 recordings on the right hand, dominant hand, or non-dominant hand were minimal, not significant, and did not lead to different conclusions.

## Results

### Accuracy

Bivariate correlations between ANS variables are presented in Table 1. Table 2 shows descriptive statistics, difference factors, LoA and outcomes of Mann-Whitney tests for ANS parameter recordings obtained from both the VU-AMS and the Empatica E4 during rest and mediation. Highly significant (all *p* < .001) and strong ICCs were observed for HR (*r* = .99), SDNN (*r* = .91), RMSSD (*r* = .89), and HF (*r* = .88). Medium yet significant ICCs were observed for RR (*r* = .62), LF (*r* = .72) and LF/HF (*r* =. 73). The difference factor for HR was particularly low with 1.60%. Differences for SDNN, LF, and HF were below 25%, those for RR, RMSSD, and LF/HF were higher than 25%. Notably, LoA were small for HR.

**Table 1:** Bivariate outcomes between ANS variables

	RR	HR	SDNN	RMSSD	LF	HF	LF/HF
RR	–						
HR	.15**	–					
SDNN	−.11**	−.58**	–				
RMSSD	−.24**	−.55**	.88**	–			
LF	.02	−.28**	.57**	.35**	–		
HF	−.03	−.41**	.76**	.83**	.00	–	
LF/HF	−.20**	.07	−.01	−.11**	−.08*	−.09*	–

Note. ANS = autonomic nervous system, SD = standard deviation, HR = heart rate, SDNN = standard deviation of the NN interval, RMSSD = root mean squared differences of successive difference of intervals, LF = low frequency, HF = high frequency, LF/HF = ratio between low and high frequency. * *p* < .05. ** *p* < .01

**Table 2:** Signal comparison of ANS parameters obtained from VU-AMS and Empatica E4 recordings (*N* = 345)

	VU-AMS		Empatica E4								
	M	SD	M	SD	DF%	ICC	CC	LoA	*U*	*p*	ES
RR	345.05	163.53	213.97	147.14	37.99	.62*	.46	−129.88 to 399.69	29,004.00	< .001	.46
HR	84.64	11.85	83.28	11.62	1.61	.99*	.60	−2.47 to 5.18	56,856.00	.115	.06
SDNN	63.24	35.16	56.94	43.08	9.96	.91*	.44	−22.40 to 32.87	54,150.00	.010	.10
RMSSD	49.99	45.75	66.28	43.08	32.59	.89*	.47	−58.06 to 23.24	36,230.00	< .001	.35
LF	1556.82	2427.98	1299.13	1658.38	16.55	.72*	.32	−3089.23 to 3528.61	54,306.50	.009	.10
HF	2126.21	4977.59	1674.06	2733.47	21.27	.88*	.33	−4998.61 to 5496.62	54,865.00	.017	.09
LF/HF	3.40	4.90	1.53	1.94	55.13	.73*	.29	−5.71 to 9.62	28,618.00	< .001	.46

Note. ANS = autonomic nervous system, CC = cross-correlation, DF% = difference factor %, ES = effect size: *r*, HF = high frequency, HR = heart rate, ICC = intraclass correlation, LF = low frequency, LF/HF = ratio between low and high frequency, LoA = Limits of Agreement, M = mean, RMSSD = root mean squared differences of successive difference of intervals, SD = standard deviation, SDNN = standard deviation of the NN interval, *U* = Mann-Whitney between groups effect size. * *p* < .01

There was no difference between VU-AMS and Empatica E4 recordings for HR. For all other parameters, significant differences were found between the VU-AMS and Empatica E4 recordings, although effect sizes were small for SDNN, LF, and HF. Differences for RR, RMSSD, and LF/HF yielded medium effect sizes. For time domain parameters, the E4 estimates SDNN lower and RMSSD higher than the VU-AMS. All frequency domain parameters estimated by the E4 were lower compared to the VU-AMS.

**Fig. 2:**
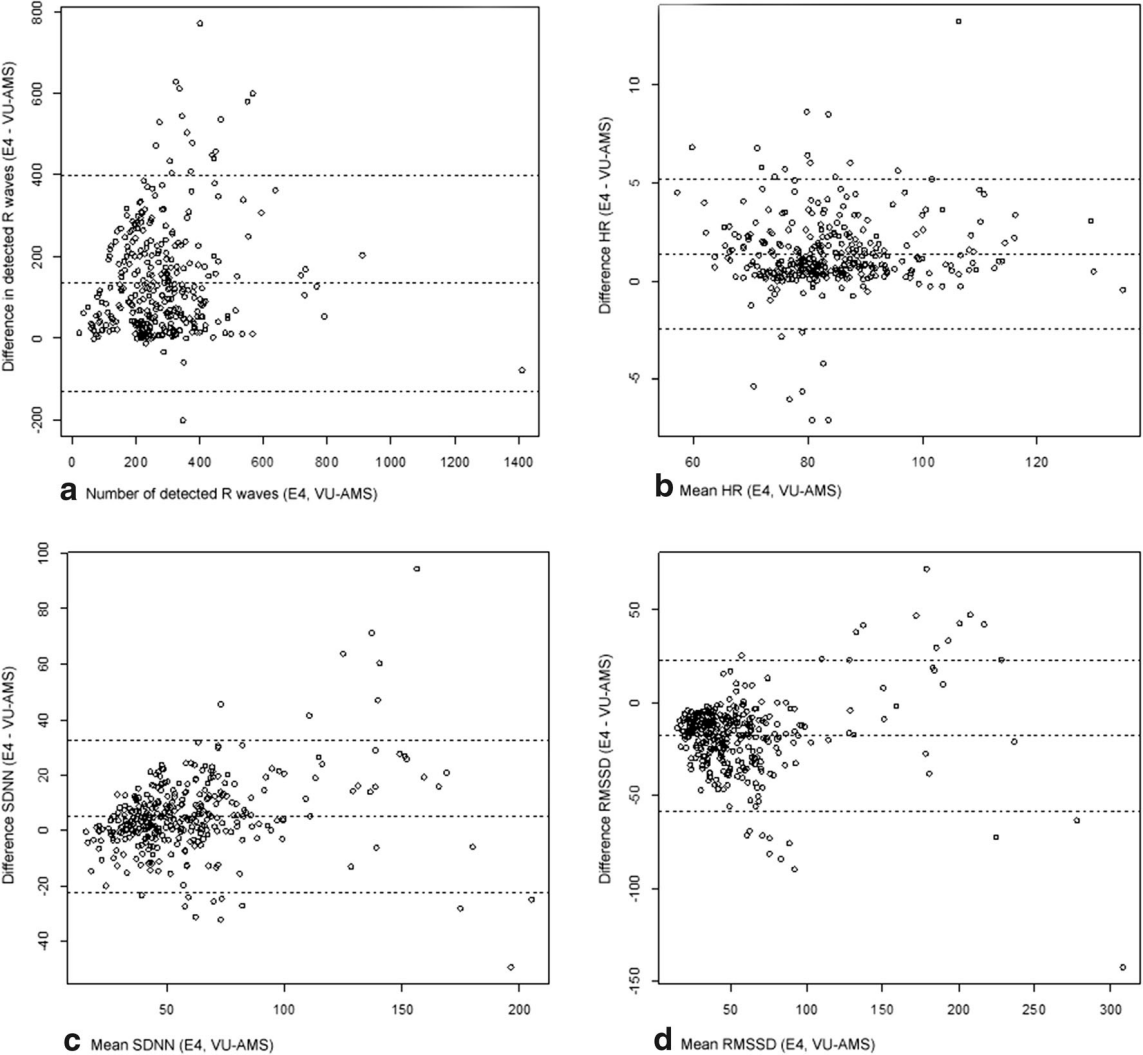
**a** to **d**: Bland-Altman Plots: Time-domain parameters. Note. HR = heart rate, RMSSD = root mean squared differences of successive difference of intervals, SDNN = standard deviation of the NN interval

**Fig. 3:**
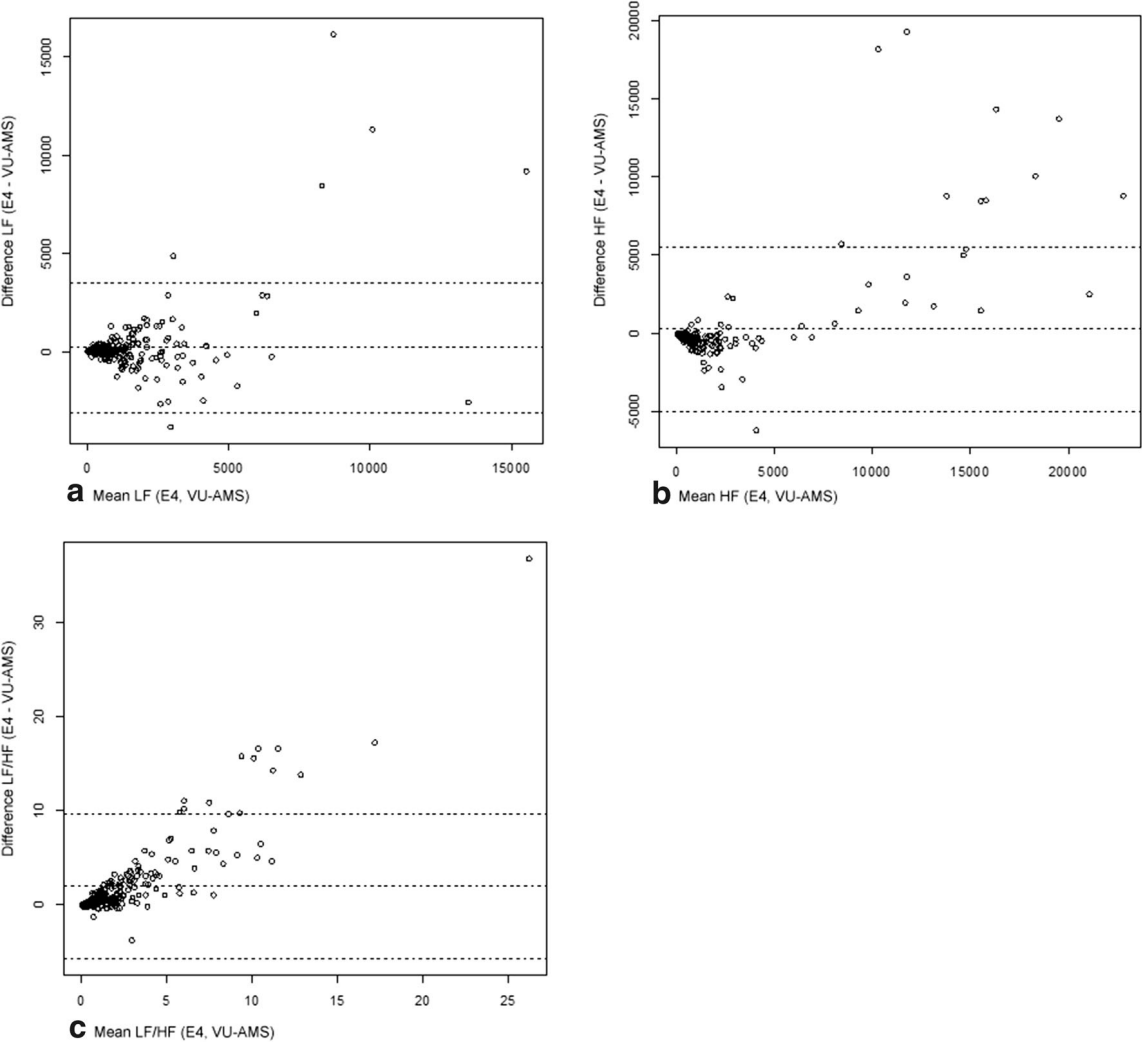
**a** to **c**: Bland-Altman Plots: Frequency-domain parameters. Note. HF = high frequency, LF = low frequency, LF/HF = ratio between low and high frequency

Figure 2A to 2D show Bland-Altman plots for combined VU-AMS and Empatica E4 recordings on the time-domain variables: (2A) RR; (2B) HR; (2C) SDNN; and (2D) RMSSD. Fig. 3A to 3C show Bland-Altman plots for combined recordings on the frequency-domain variables: (3A) LF; (3B) HF; and (3C) LF/HF. The differences between and the average of the two measures are represented on the Y-axis and X-axis, respectively.

### Predictive Value

Table 3 shows the descriptive statistics for both resting and meditation ANS parameters per game (*Muse, Daydream, Wild Divine*). Separately for each game, Mann-Whitney tests were conducted to test whether there was a difference in HR, SDNN, RMSSD, LF, HF, and LF/HF between resting and meditation ANS parameters. Based on the significant differences, for all parameters but RMSSD, testing outcomes of Empatica E4 recordings led to the same conclusions as for testing outcomes of VU-AMS recordings.

**Table 3:** Differences between resting and meditation ANS parameters obtained from the VU-AMS and Empatica E4 per condition

	Baseline ANS				ANS during meditation				Testing for differences			
	VU-AMS		Empatica E4		VU-AMS		Empatica E4		VU-AMS		Empatica E4	
	*M*	*SD*	*M*	*SD*	*M*	*SD*	*M*	*SD*	*U*	*p*	*U*	*p*
HR												
Muse	89.98	12.25	88.44	12.51	85.36	12.94	83.79	12.18	3449.00	< .001	3435.5	.002
Daydream	88.83	7.09	86.74	7.70	84.90	7.59	82.87	7.88	444.00	.012	423.50	.008
Wild Divine	77.50	9.50	75.92	9.93	75.73	7.43	75.28	8.30	595.00	.597	559.00	.995
SDNN												
Muse	49.45	18.49	45.85	17.03	49.37	15.34	46.68	13.53	4794.00	.881	4358.00	.420
Daydream	43.22	15.50	41.82	15.97	56.97	16.11	52.89	16.71	325.00	< .001	412.50	.007
Wild Divine	104.94	43.64	95.09	49.34	119.37	38.34	99.23	31.42	488.00	.086	445.50	.155
RMSSD												
Muse*	29.54	15.97	54.09	23.82	37.27	19.19	54.69	22.74	3652.00	.001	4555.50	.756
Daydream*	27.47	13.06	51.03	21.10	37.61	14.41	59.15	18.42	368.00	.001	506.00	.089
Wild Divine	110.55	74.30	121.88	84.30	109.25	62.25	105.71	56.59	638.00	.954	524.00	.662
LF												
Muse	915.34	889.27	802.51	766.86	821.87	684.10	760.74	722.40	1688.00	.986	4615.00	.838
Daydream	532.34	394.17	633.43	609.70	1254.81	1019.76	1392.24	1295.98	283.00	< .001	369.00	.001
Wild Divine	1695.82	889.49	1778.18	1124.74	5840.89	5071.62	4051.13	2945.14	506.00	< .001	253.00	< .001
HF												
Muse	345.65	405.47	663.57	613.32	581.32	599.53	919.47	867.93	1175.00	.005	3672.50	.010
Daydream	371.61	373.80	694.93	645.88	755.15	754.40	1172.21	980.27	367.00	.004	444.00	.016
Wild Divine	8259.74	9113.69	5093.93	5191.74	7051.98	7527.71	4792.88	4401.17	357.00	.569	547.00	.877
LF/HF												
Muse	5.63	7.28	1.74	1.82	3.08	4.03	1.42	1.71	1184.00	.006	3906.50	.049
Daydream	2.10	1.56	1.11	0.68	2.40	1.80	1.42	0.96	530.00	.328	561.00	.272
Wild Divine	0.69	0.68	0.60	0.51	3.75	4.30	2.68	3.69	234.00	.008	354.00	.008

Note. *ANS* autonomic nervous system, *SD* standard deviation, *HR* heart rate, *SDNN* standard deviation of the NN interval, *RMSSD* root mean squared differences of successive difference of intervals, *LF* low frequency, *HF* high frequency, *LF/HF* ratio between low and high frequency. * = different testing outcomes based on VU-AMS and Empatica E4 recordings

## Discussion

### Key findings

The present study was conducted to evaluate the accuracy and predictive value of the Empatica E4 wristband by comparing it to the gold standard VU-AMS in a clinical population of adolescents in residential care. As for accuracy, results show that Empatica E4 recordings of HR are highly comparable to VU-AMS recordings. For the other parameters, significant differences were found, although effect sizes were small for SDNN, LF, and HF. The Empatica E4 has good predictive value for all ANS parameters except for RMSSD. The statistical tests indicated that the results of the Empatica E4 and VU-AMS were comparable in distinguishing between resting and meditation.

The Empatica E4 performs excellent in estimating HR. Empatica uses two algorithms to detect heartbeats based on the blood volume pulse. Empatica [34] states that their goal is to only detect beats of which they are certain. As a result of movement, pressure, or not wearing the device tight enough, the E4 fails to detect all beats resulting in data loss, and hence, misses the IBI on which the more complex calculations of HRV parameters are based. This loss of data resulted in the relatively large difference (37.5%) in RR detection between the Empatica E4 and the VU-AMS. This is comparable with other studies, for example, Van Lier et al. [19] reported an artefact percentage of 45% in their data.

Yet, the results indicate that in situations where participants show minimal movement, as in our study, Empatica E4 recordings of HR and SDNN are highly accurate, although the Empatica E4 recordings are probably a slight underestimation of the real SDNN values (given that the VU-AMS provides higher, and presumbly more accurate, values). Surprisingly, the RMSSD recordings, seem unreliable, since these not only differ substantially from the VU-AMS values, but also lead to different outcomes of statistical tests. Regarding the frequency-domain parameters, LF and HF perform most promising with minor differences from the VU-AMS recordings.

### Comparison to other studies

Zheng and Poon [20] and McCarthy et al. [8] did not provide any parameters besides heart rate. Like Ollander et al. [9], we calculated difference factors as an estimation of the difference between recordings of the two devices. Similar to their results, in our study difference factors for time domain parameters were very low for HR and higher for the time domain parameter RMSSD. Unfortunately, they did not report SDNN. Regarding the frequency domain parameters, our results for LF were comparable, but our DF% was lower for HF and higher for LF/HF. It should be noted that their sample was very small, so no strong inferences about their findings can be drawn.

Of all previous studies, Van Lier et al. [19] provided the most extensive validation. Unfortunately, for time domain parameters, they only reported RMSSD and means and SDs for the RR intervals. Although they reported that data of the Empatica E4 can be considered valid for HR and RMSSD, we cannot make a comparison on SDNN, another value besides HR that we considered as very promising. Regarding validity on parameter level, our findings with respect to HR show – in line with findings of Ollander et al. [9], McCarthy et al. [8], [20], and Van Lier et al. [19] – that the Empatica E4 suited for estamating HR.

When we compare our results to the Polar validation studies of Giles et al. [11] and Caminal et al. [10], it can be noted that our correlations – although significant – are lower than the correlations of the Polar V800 and ECG recordings. These studies did not report mean HR, but for all other parameters, both time and frequency domain, the LoA reported in our study were wider. However, although these studies did use ECG to compare the Polar V800 to, these were not gold standard devices such as the VU-AMS or the Biopac.

### Empatica E4 removal of artefacts

The PPG sensor of the Empatica E4 has LEDs that produce light oriented towards the skin. The light receiver measures the portion of the light that is reflected back. Therefore, the sensor requires direct contact with the skin and is sensitive to motion artefacts and incorrect placement [35, 36]. The Empatica E4 automatically removes these artefacts from the data, which results in shorter recordings. We found a difference score of approximately 40% in recording time between the VU-AMS and the Empatica E4, although there was minimal movement during the recordings and Empatica states that measurements in static condition could use IBI data as provided [37]. The large amount of missing IBI data suggests that the Empatica E4 is highly sensitive to motion and motion artefacts, which impedes in particular its applicability for long-term recordings in daily life and experimental conditions that include exercise or movement. Artefacts in real-life situations are expected to have a significant influence on parameter estimation, which warrants further research on wearable, wrist-worn devices.

### Strengths and limitations

Although four previous studies have provided a preliminary examination of the Empatica E4, this is, to our knowledge, the first study examining the validity of the Empatica E4 wristband while worn on both wrists and compared with a gold standard ECG device. The study was conducted with fifteen participants, but due to the repeated recording moment, our sample for time-domain analysis included 345 recording segments, which can be considered a valid sample size to validate ANS parameters [19]. Moreover, this study was conducted in a clinical population of adolescents in residential care and thus requires minimal translation to be relevant for clinical care. While posing substantial scientific challenges, research in clinical contexts is critical for practical innovation. We need to be aware of both the practical advantages and limitations of wearable HRV monitoring devices to decide whether these devices can be used in clinical care. For example, it should be noted that halfway the study, two participants refused to continue with the VU-AMS recordings due to discomfort, while they were willing to complete the remaining sessions wearing only the Empatica E4 wristbands. This illustrates the major practical advantage of wearable monitoring devices: wristbands do not require the application of electrodes and are non-intrusive, comfortable, and easy to wear.

To conduct the analyses for this study, we used data from a feasibility study that focused on measuring HR and HRV. While the Empatica E4 also measures EDA, XYZ raw acceleration, and skin temperature, the available data did not include these parameters. In particular EDA is a useful measure of sympathetic activation [38]. We have to refrain from drawing strong conclusions regarding the validity of the Empatica E4 only based on its HR and HRV data. Future validation studies should include assessments of the other parameters provided by the Empatica E4, and possibly combine information from different parameters to see whether combinations could be even more informative. Also, our recordings were made under static conditions while participants were at rest. While informative as a first step toward validation of the Empatica E4, future research that include gold a standard reference device could focus on its ability to distinguish between states of stress and states of rest, and its recording quality when participants do not sit still. As our measurements did not include a stressor that was expected to prompt physiological changes, we were unable to assess validity on the event level.

In this validation study we used Kubios to process the Empatica E4 recordings, as recommended by Empatica [39]. For the VU-AMS recordings, we used the DAMS program that was developed to analyze VU-AMS recordings (Vrije [32]). The reported differences between the Empatica E4 and VU-AMS recordings may – partly – be caused by software differences in processing and calculating HR and HRV parameters. In particular for frequency domain parameters, the use of different mathematical methods could lead to different results [40]. It is noteworthy that in this study, the Empatica E4 performed worst on the frequency domain parameters. Although it is possible to analyze VU-AMS recordings in Kubios, we decided not to since this would deviate from the gold standard method that we wanted to compare the Empatica E4 to. Agreement between the two devices might have been higher when VU-AMS recordings were also analyzed with Kubios.

## Conclusions

The development of wearable health technology provides new opportunities to measure HRV with easy-to-use devices such as the Empatica E4 wristband in clinical practice. Findings of the present study indicate that the Empatica E4 is practical and feasible for recording a limited set of ANS parameters. The strong correlations and agreement found between Empatica E4 and VU-AMS recordings for mean HR and SDNN suggest its potential as a valid tool for research on HR and HRV while people are at rest. While more research needs to be conducted, this study could be considered as a first step to support the use of HRV recordings provided by wearables.

## Data Availability

Data are not available. Since these data were conducted in a clinical sample, the ethics committee required limited data access.

## Abbreviations

ANS: autonomic nervous system
CC: cross-correlations
ECG: electrocardiography
HF: high frequency
HR: heart rate
HRV: heart rate variability
LF: low frequency
LF/HF: ratio between low and high frequency
NN: normal-to-normal
PNS: parasympathetic nervous system
SD: standard deviation
SDNN: standard deviation of the NN interval
SNS: sympathetic nervous system
RMSSD: root mean squared differences of successive difference of intervals
